# Altered leptin level in autism spectrum disorder and meta-analysis of adipokines

**DOI:** 10.1186/s12888-024-05936-4

**Published:** 2024-07-01

**Authors:** Lei Chen, Li-Ming Liu, Mei Guo, Yang Du, Yue-wen Chen, Xi-Yue Xiong, Yong Cheng

**Affiliations:** 1 Key Laboratory of Ethnomedicine of Ministry of Education, Center On Translational Neuroscience, School of Pharmacy, Minzu University of China, 27 South Zhongguancun Avenue, Beijing, 100081 China; 2 Institute of National Security, Minzu University of China, Beijing, China; 3 NHC Key Laboratory of Birth Defect for Research and Prevention, Hunan Provincial Maternal and Child Health Care Hospital, Changsha, 410008 China; 4 Chinese Academy of Sciences Key Laboratory of Brain Connectome and Manipulation, The Brain Cognition and Brain Disease Institute, Shenzhen Key Laboratory of Translational Research for Brain Diseases, Shenzhen Institute of Advanced Technology, Chinese Academy of Sciences, Shenzhen–Hong Kong Institute of Brain Science–Shenzhen Fundamental Research Institutions, Shenzhen,, 518055 Guangdong China; 5 Guangdong Provincial Key Laboratory of Brain Science, Disease and Drug Development, HKUST Shenzhen Research Institute, Shenzhen, 518057 Guangdong China

**Keywords:** Adipokines, Leptin, Autism spectrum disorder, Meta-analysis

## Abstract

**Background:**

Increasing evidence suggests that leptin is involved in the pathology of autism spectrum disorder (ASD). In this study, our objective was to investigate the levels of leptin in the blood of children with ASD and to examine the overall profile of adipokine markers in ASD through meta-analysis.

**Methods:**

Leptin concentrations were measured using an enzyme-linked immunosorbent assay (ELISA) kit, while adipokine profiling, including leptin, was performed via meta-analysis. Original reports that included measurements of peripheral adipokines in ASD patients and healthy controls (HCs) were collected from databases such as Web of Science, PubMed, and Cochrane Library. These studies were collected from September 2022 to September 2023 and followed the Preferred Reporting Items for Systematic Reviews and Meta-Analyses (PRISMA) guidelines. Standardized mean differences were calculated using a random effects model for the meta-analysis. Additionally, we performed meta-regression and explored heterogeneity among studies.

**Results:**

Our findings revealed a significant increase in leptin levels in children with ASD compared to HCs (*p* = 0.0319). This result was consistent with the findings obtained from the meta-analysis (*p* < 0.001). Furthermore, progranulin concentrations were significantly reduced in children with ASD. However, for the other five adipokines analyzed, there were no significant differences observed between the children with ASD and HCs children. Heterogeneity was found among the studies, and the meta-regression analysis indicated that publication year and latitude might influence the results of the meta-analysis.

**Conclusions:**

These findings provide compelling evidence that leptin levels are increased in children with ASD compared to healthy controls, suggesting a potential mechanism involving adipokines, particularly leptin, in the pathogenesis of ASD. These results contribute to a better understanding of the pathology of ASD and provide new insights for future investigations.

**Supplementary Information:**

The online version contains supplementary material available at 10.1186/s12888-024-05936-4.

## Introduction

Autism spectrum disorder (ASD) is a neurodevelopmental disorder characterized by early impairments in social communication and interaction, as well as restricted and repetitive behaviors [1, 2]. It affects approximately 1% of the global population, with a higher prevalence in males than in females [3]. Both genetic and environmental factors have been suggested to contribute to the development of ASD [4].

Recent studies have associated elevated plasma leptin levels in early childhood with a higher incidence of autism, as observed in the Boston Birth Cohort [5]. Leptin, originally known for its role in regulating food intake and energy balance [6], has gained increased attention in the field of psychiatry [7]. Leptin is in the same protein family as interleukin (Il)-6, an inflammatory cytokine, and its dysregulation has been associated with psychopathology, and evidence suggests that this relationship is related to leptin’s inflammatory function [8–10]. It has been implicated in various psychiatric disorders such as schizophrenia [11, 12], major depressive disorder [13], and bipolar disorder [14, 15]. The role of leptin in ASD has also become a subject of interest, but the underlying mechanisms remain to be determined. Other adipokines, which are inflammatory mediators mainly secreted by adipose tissue and some immune cells, have also garnered increasing attention in ASD research.

Adipokines have the potential to impact immune responses and are believed to play a role in the pathophysiology of ASD [16]. Several studies have reported alterations in adipokine levels in ASD, including downregulation of adiponectin [17], among others. However, the findings regarding adipokines in ASD are inconsistent, possibly due to differences in patient populations, variations in methodologies, or small sample sizes lacking statistical power.

To address these discrepancies and clarify the relationship between adipokines and autism, this study aimed to investigate serum leptin levels in ASD patients and HCs, and to conduct a systematic review of the literature. This comprehensive review, the first of its kind, will assess studies that have measured adipokines in both ASD patients and control groups. By doing so, we hope to identify potential differences in adipokine profiles associated with the development of ASD and shed light on the possible involvement of adipokines in the pathogenesis of autism.

## Material and method

### Subjects and sample collection

**Fig. 1:**
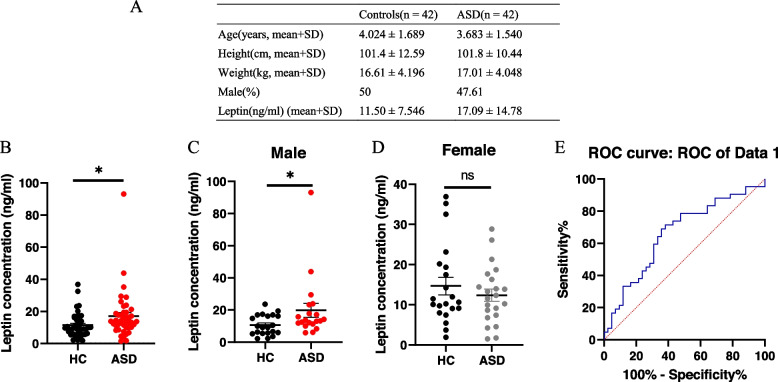
Serum leptin levels in ASD patients and HC subjects. **A** Demographic and clinical characteristics of patients and controls. **B** Serum leptin levels in ASD patients and HC subjects. **C** Serum leptin levels in male ASD patients and male HC subjects. **D** Serum leptin levels in female ASD patients and female HC subjects. **E** ROC curves evaluating the accuracy of serum leptin levels in differentiating between ASD patients and HC subjects. * *p* < 0.05, ** *p* < 0.01, *** *p* < 0.001

A total of 42 patients with autism spectrum disorder (ASD) were recruited from Hunan Provincial Maternal and Child Healthcare Hospital. The diagnosis of ASD was made according to the Diagnostic and Statistical Manual of Mental Disorders, Fifth Edition. Additionally, 42 healthy control (HC) subjects without psychiatric illnesses, as evaluated by trained psychiatrists, were also recruited from the same hospital via advertisement. The age of the enrolled children was between 3–7 years old, and there was no other medical history. Autistic children with a history of seizure or psychotic disorders, severe head injury, or any other acute or chronic mental disease, inflammatory illness, or comorbidities were excluded. Control participants with a history of any immunological or infectious illness; or metabolic, psychiatric, or neurological disorders were also excluded. The demographic and clinical characteristics of the patients and controls are presented in Fig. 1A. Written informed consent was obtained from all participants, and the study protocol was approved by the Ethics Committee at Minzu University of China, adhering to the principles of the Declaration of Helsinki. Peripheral blood was collected from all participants in the morning, between 7 and 8 AM. Blood samples were collected in anticoagulation tubes, and serum samples were obtained by centrifugation at 5,000 rpm for 15 min. The samples were stored at -80 °C until further analysis.

### Leptin concentration measurement

Leptin levels in peripheral blood were measured using a commercially available ELISA kit (Wuhan Eiaab Science Incorporated Company, Wuhan, China), following the manufacturer's instructions.

### Article selection process

This study followed the Preferred Reporting Items for Systematic Reviews and Meta-Analyses guidelines [18]. Two investigators systematically searched PubMed, Web of Science, and Cochrane Library from September 2022 to September 2023. The search was conducted using specific keywords related to autism spectrum disorder and adipokines: (autism or autistic disorder or ASD) and (leptin or ghrelin or adiponectin or soluble leptin receptor or lipocalin or resistin or insulin or omentin or obestatin or osteopontin or chemerin or visfatin or vaspin or apelin or progranulin or dipeptidyl peptidase 4 or retinol-binding protein 4 or adipokines). Only peer-reviewed English articles published between January 1971 and January 2023 were included for analysis. The inclusion criteria involved studies that provided serum or plasma adipokine data for children with ASD and controls. Studies were excluded if they lacked necessary data, involved animal models, lacked a healthy control group, used samples other than serum or plasma, or did not involve patients with ASD.

### Data extraction

Data extraction included potential adipokine concentrations with standard deviation (SD), sample size, and *P* values for effective size (ES) generation. Information such as country, gender, age, sampling source, publication year, assay type, and diagnosis were also recorded for each included study (eTable 1). The quality of the included studies was assessed using the Newcastle–Ottawa Quality Assessment Scale (eTable 2).

### Data analysis

Comprehensive Meta-analysis software was used to analyze the extracted data. When necessary, sample size and *P* values were utilized for ES generation due to a lack of direct data on adipokine concentrations. The random-effects model was employed when significant between-study heterogeneity was present, while the fixed-effect model was used otherwise. Hedge's g statistic analysis, sensitivity analysis, I2 statistic, Cochrane Q test, meta-regressions, and Egger's test were performed as previously described [19]. Statistical significance was set at *P* < 0.05, except for the Cochrane Q test, where *P* < 0.10 was considered statistically significant.

## Results

### Investigation of serum leptin concentrations in ASD patients

The demographic and clinical characteristics of patients and controls are presented in Fig. 1A. The ELISA assay revealed that serum leptin levels were significantly increased in ASD patients compared to HC subjects (Mean difference = 5.589, 95% confidence interval (CI) of difference = 0.4946 to 10.68, *p* = 0.0319) (Fig. 1B). Further analysis showed a significant reduction in serum leptin levels in male ASD patients (Mean difference = 9.194, 95% CI of difference = 0.2013 to 18.19, *p* = 0.0453) (Fig. 1C), while no significant difference was observed in female ASD patients (Mean difference = -2.270, 95% CI of difference = -7.607 to 3.068, *p* = 0.3955) (Fig. 1D). Receiver Operating Characteristic curve (ROC) analysis indicated that leptin demonstrated limited accuracy in differentiating between ASD patients and controls, with an area under curve (AUC) of 0.660 (95% CI: 0.542—0.778) (Fig. 1E).

### Meta-analysis of adipokines

**Fig. 2:**
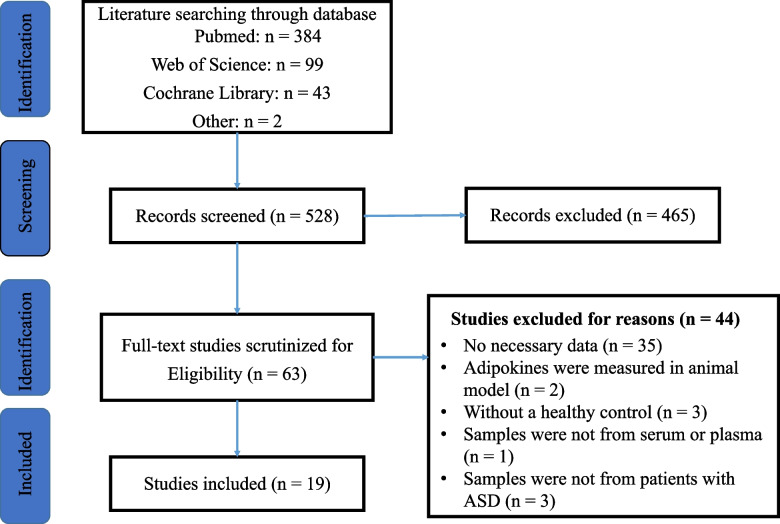
PRISMA flowchart depicting the literature search process

After thorough screening, a total of 19 articles were included in the meta-analysis, providing data on 2196 participants, including 740 ASD subjects and 1456 healthy controls. The distribution of the literature selection process is presented in Fig. 2.

### Differences in peripheral marker concentrations

**Fig. 3:**
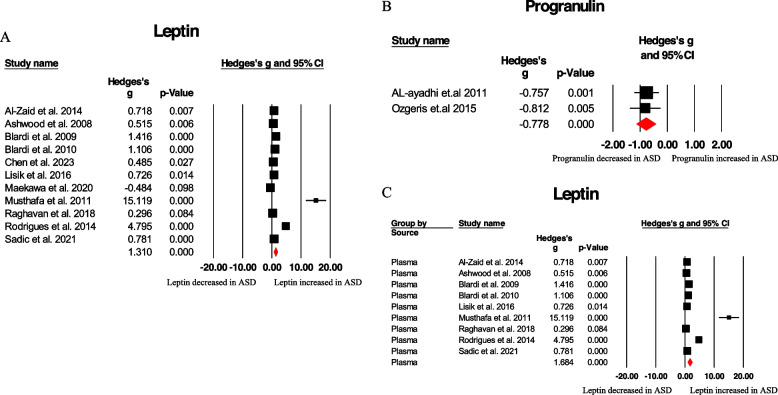
Forest plots illustrating the effect sizes for leptin (**A**) and progranulin (**B**) in ASD patients compared to HC subjects. Forest plot demonstrating pooled results and heterogeneity analysis for leptin levels between ASD patients and HC subjects, stratified by source of sampling (plasma). Heterogeneity: Q = 131.262, *p* < 0.001, I2 = 93.905, 9 studies

**Fig. 4:**
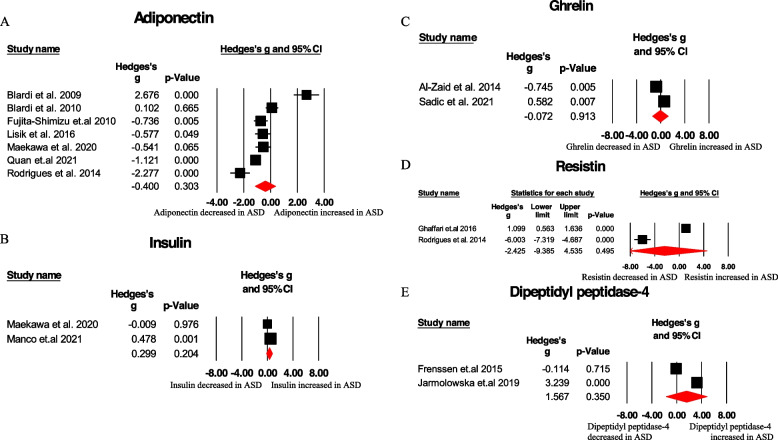
Forest plots illustrating the effect sizes for adiponectin (**A**), insulin (**B**), ghrelin (**C**), resistin (**D**) and dipeptidyl peptidase-4 (**E**) in ASD patients compared to HC subjects

Significant differences in peripheral adipokine concentrations were observed between ASD and HC groups for two analytes. Leptin levels in the blood were significantly higher in ASD children compared to HC children (Hedges g (95% CI): 1.310 (0.684 to 1.936), Q = 149.362, *p* < 0.001, 11 studies) (Table 1 and Fig. 3A). Conversely, progranulin concentrations were significantly decreased in ASD children compared to controls (Hedges g (95% CI): -0.778 (-1.130 to -0.427), Q = 0.022, *p* = 0.882, 2 studies) (Table 1 and Fig. 3B). However, adiponectin, insulin, ghrelin, resistin, and dipeptidyl peptidase-4 did not show significant associations with ASD (Table 1). Further investigation of heterogeneity was conducted for the leptin analysis due to the limited number of studies available for progranulin. High levels of heterogeneity were observed in the plasma leptin analysis (I2 = 93.905; 9 articles) (Fig. 3C). However, adiponectin, insulin, ghrelin, resistin and dipeptidyl peptidase-4 did not show the significantly difference in ASD children compared to HC children (Fig. 4A-E), which may require further researches.

**Table 1:** Summary of Comparative Outcomes for Measurements of Blood Adipokines Levels

Marker	No.of Studies	No.With ASD/Controls	Main Effect			Heterogeneity				Publication Bias	
			Hedges *g* (95% CI)	*Z* Score	*P* Value	*Q* Statistic	*df*	*P* Value	*I*^*2*^ Statistic	Egger Intercept	*P* Value
Leptin	11	367/919	1.310 (0.684 to 1.936)	4.100	< .001	149.362	10	< .001	93.305	8.07355	0.00178
Progranulin	2	60/75	-0.778 (-1.130 to -0.427)	-4.345	< .001	0.022	1	0.882	0	/	/
Adiponectin	7	244/239	-0.400 (-1.160 to 0.361)	-1.030	0.303	86.318	6	< .001	93.049	4.99206	0.32005
Insulin	2	81/266	0.299 (-0.162 to 0.759)	1.270	0.204	2.274	1	0.132	56.022	/	/
Ghrelin	2	75/72	-0.072 (-1.372 to 1.228)	-0.109	0.913	14.988	1	< .001	93.328	/	/
Resistin	2	60/49	-2.425 (-9.385 to 4.535)	-0.683	0.495	95.908	1	< .001	98.957	/	/
Dipeptidyl peptidase-4	2	104/73	1.567 (-1.719 to 4.853)	0.935	0.350	67.572	1	< .001	98.520	/	/

*Abbreviations: df* Degrees of freedom, *ASD* Autism spectrum disorder

### Investigation of heterogeneity

**Fig. 5:**
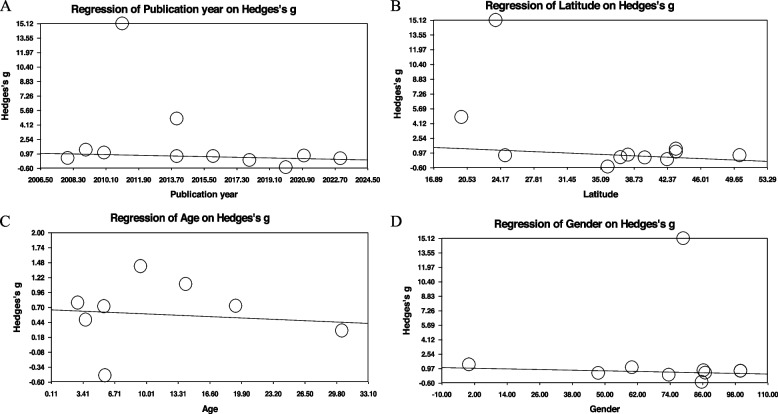
Meta-regression analysis examining the relationship between leptin levels and publication year (**A**), latitude (**B**), age (**C**), and gender (**D**). Association between publication year (Regression coefficient [SE]: -0.0399 [0.0147], 95% CI: -0.0687 to -0.0112, *p* = 0.0065), latitude (Regression coefficient [SE]: -0.0409 [0.0123], 95% CI: -0.0650 to -0.0167, *p* = 0.0009), age (Regression coefficient [SE]: -0.0071 [0.0079], 95% CI: -0.0226 to 0.0083, *p* = 0.3651), and gender (Regression coefficient [SE]: -0.0058 [0.0035], 95% CI: -0.0127 to -0.0011, *p* = 0.0972) with effect size (Hedges's g) for leptin levels

Meta-regression analysis indicated a significant association between publication year and leptin concentrations (*p* = 0.0065) (Fig. 5A). Additionally, latitude was found to be a confounding factor in the regression analysis (*p* = 0.0009) (Fig. 5B). However, age and gender did not demonstrate a significant effect on the study outcomes (Fig. 5C, D). No evidence of publication bias was detected through funnel plots, Egger's test, or trim and fill tests (eFigure).

## Discussion

This study examined the significant increase in serum leptin levels in ASD patients compared to healthy controls. Additionally, a meta-analysis of 19 studies with a total sample size of 740 patients and 1456 controls revealed alterations in leptin and progranulin levels in individuals with ASD. The analysis highlights that the significant difference in leptin levels primarily focuses on plasma and suggests a potential influence of publication year and latitude on the observed heterogeneity. It is important to note that the results for progranulin present limitations, requiring further studies for confirmation.

It is widely recognized that children with ASD have a higher risk of overweight or obesity compared to those with typical development [20–22]. This study explored the role of leptin, an adipocyte-derived factor, in ASD and its potential involvement in the development of the disorder. While previous research supports the critical role of leptin as a regulator of food intake and energy expenditure, recent studies have proposed that dysregulation of leptin is a mechanism for psychopathologies, including ASD [23]. This proposal is based on the observed comorbidity between obesity and various mental illnesses [24]. Moreover, inflammation is posited to be a potential mechanism through which leptin may impact ASD, given its pro-inflammatory cytokine properties [25, 26]. Additionally, altered leptin levels have been associated with structural and functional brain changes, including lower brain weight, reduced myelin content, neuronal soma size, and synaptic protein levels [27–29]. Notably, studies have observed a higher concentration of leptin in the anterior cingulate gyrus of individuals with autism compared to controls [30]. Furthermore, leptin has been found to suppress serotonin synthesis, suggesting another potential biological pathway through which leptin may impact ASD [31].

As brain samples are challenging to obtain, researchers often examine peripheral leptin levels to elucidate on the underlying mechanisms involved. A study comparing 70 children diagnosed with ASD to 99 age-matched control children reported a significant increase in leptin levels in autism, with higher levels observed in early onset cases compared to regressive autism [32]. Similarly, another study demonstrated increased leptin levels in autistic patients over a one-year period [23]. Consistent with these findings, our study also revealed a significant increase in leptin levels in ASD patients compared to healthy controls. Furthermore, we analyzed the relationship between gender and leptin concentration, finding higher leptin levels in boys with ASD compared to healthy boys, while no significant difference was observed in girls. Our results, combined with the meta-analysis findings, highlight the potential of leptin as an important focus in ASD research. However, our ROC curve did not exhibit high specificity or sensitivity for leptin, likely due to the small sample size. Although elevated peripheral leptin may not serve as a unique biomarker for ASD, the consistent increasing trend in ASD patients provides confidence in its potential significance. To explore changes in other adipokines, we conducted a meta-analysis, which revealed decreased levels of progranulin in ASD compared to control, while other adipokines did not exhibit significant differences. This lack of significant findings may be attributed to the limited research on adipokines in the context of ASD, emphasizing the need for further studies to identify multiple factors and establish new diagnostic approaches for ASD.

Although the significant results remained robust when considering factors such as age, IQ, BMI, and medication status, heterogeneities were observed among studies investigating leptin in ASD [32]. The sampling source (serum or plasma) did not account for these heterogeneities. Meta-regressions conducted in our study indicate that publication year and latitude significantly influence the associations between leptin and ASD. This analysis partially addresses the between-study heterogeneities through subgroup and meta-regression analyses, which strengthens the present meta-analysis. Moreover, it is important to acknowledge that other adipokines can also modulate brain functions [33]. Previous research has shown changes in the levels of ghrelin and adiponectin in ASD children [17, 34]. However, the limited research investigating the relationship between ASD and adipokines poses challenges in explaining the underlying mechanisms involved. In our study, we identified only two studies reporting changes in progranulin levels in ASD patients, underscoring the need for increased attention to adipokines in future research endeavors.

Despite these findings, it is essential to acknowledge the inherent limitations of this study. The first limitation is the small sample size, although the meta-analysis approach helps to confirm the trend of leptin level changes. The second limitation is the scarcity of studies focusing on adipokines in the context of ASD, highlighting the need for further development in future meta-analyses. Last, while this study investigated changes in adipokine concentrations in the peripheral blood of ASD children, the extent to which these measurements reflect brain activity remains unknown, despite the potential involvement of leptin resistance mechanisms [35].

## Conclusion

Our study demonstrated a significant increase in peripheral blood leptin levels in ASD patients compared to healthy controls. Additionally, a meta-analysis involving 19 studies provided evidence of altered leptin levels in ASD. Furthermore, progranulin exhibited a contrasting trend, suggesting the significant potential of adipokines as biomarkers for ASD, albeit requiring further analysis. Future investigations into adipokines, particularly leptin, as potential diagnostic markers and therapeutic targets for ASD are essential.

### Supplementary Information

Supplementary Material 1.

## Data Availability

Data and materials are available on request. The email of corresponding author is yongcheng@muc.edu.cn.
