# Supplementary material for: Altered leptin level in autism spectrum disorder and meta-analysis of adipokines
Source: BMC Psychiatry. 2024 Jul 1;24:479. doi: 10.1186/s12888-024-05936-4 (PMC11218410; doi:10.1186/s12888-024-05936-4)
Supplement: Supplementary file 1 — Supplementary Material 1. [file 12888_2024_5936_MOESM1_ESM.docx]

# Supplementary Online Content

**eTable 1:** Characteristics of included studies measuring adipokines.

**eTable 2:** Newcastle-Ottawa quality assessment scale for included studies.

**eFigure:** Funnel plots for leptin.

# eReference: Included 19 studies for meta-analysis

**eTable 1: Characteristics of included studies measuring adipokines**

| **Study/Year** | **Markers Measured** | **Country** | **Samples (ASD/HC)** | **Gender (%Male) (ASD/HC)** | **Mean Age** **(ASD/HC)** | **Age/Gender matched** | **Diagnosis** | **Sample Source** | **Assay type** | **BMI**  **(ASD/HC)** | **Sources of healthy volunteers** |
| --- | --- | --- | --- | --- | --- | --- | --- | --- | --- | --- | --- |
| AL-ayadhi et.al 2011 | progranulin | Riyadh, Saudi Arabia | 40/40 | 80/82.5 | 7.98/7.83 | Y/Y | DSM-IV | Plasma | ELISA | NA | Hospital |
| Al-Zaid et al. 2014 | ghrelin; leptin | Riyadh, Saudi Arabia | 31/28 | 100/100 | 5.59 /5.44 | Y/Y | DSM-IV; ADOS | Plasma | ELISA | 15.9/15.3 | NA |
| Ashwood et al. 2008 | leptin | California, USA | 70/50 | 87/76 | NA | Y/N | DSM-IV; ADOS; ADI-R | Plasma | ELISA | NA | NA |
| Chen et al. 2023 | leptin | Beijing, China | 42/42 | 47.6/50 | 3.68/4.02 | Y/Y | DSM-IV | Serum | ELISA | NA | Hospital |
| Blardi et al. 2009 | leptin; adiponectin | Siena, Italy | 16/16 | 0/0 | 9.4 /9.9 | Y/Y | NA | Plasma | Kits | NA | Primary Health Care Centers |
| Blardi et al. 2010 | leptin; adiponectin | Siena, Italy | 35/35 | 60/60 | 14.1/14.1 | Y/Y | DSM-IV; ADOS | Plasma | Kits | NA | Hospital |
| Frenssen et.al 2015 | dipeptidyl peptidase-4 | Antwerp, Belgium | 18/22 | NA | 16.22/16.00 | Y/N | DSM-IV | Serum | Colorimetric method | 20.06/20.32 | School |
| Fujita-Shimizu et.al 2010 | adiponectin | Shizuoka, Japan | 31/31 | 100/100 | 11.6/12.1 | Y/Y | DSM-IV-TR | Serum | ELISA | 17.7/17.8 | Local community |
| Ghaffari et.al 2016 | resistin | Ahvaz, Iran | 30/30 | 73.3/73.3 | 7.72/8.4 | Y/Y | DSM-IV-  TR | Serum | ELISA | 16.15/16.19 | Local community |

| **Study/Year** | **Markers Measured** | **Country** | **Samples (ASD/HC)** | **Gender (%Male) (ASD/HC)** | **Mean Age**  **(Years)** **(ASD/HC)** | **Age/Gender matched** | **Diagnosis** | **Sample Source** | **Assay type** | **BMI**  **(ASD/HC)** | **Sources of healthy volunteers** |
| --- | --- | --- | --- | --- | --- | --- | --- | --- | --- | --- | --- |
| Jarmołowska et.al 2019 | dipeptidyl peptidase-4 | Olsztyn, Poland | 86/51 | 82.6/62.7 | 5.4/5.2 | Y/N | ICD-10 | Serum | ELISA | NA | NA |
| Lisik et al. 2016 | leptin; adiponectin | Katowice, Poland | 23/24 | 100/100 | 19.3/21.8 | Y/Y | DSM-IV | Plasma | ELISA | 26.5/22.9 | School |
| Maekawa et al. 2020 | leptin; adiponectin; insulin | Japan | 21/26 | 85.7/53.8 | 5.70/6.05 | Y/N | DSM-IV-TR; ADI-R | Serum | ELISA | 14.95/15.75 | Local community |
| Manco et.al 2021 | insulin | Rome, Italy | 60/240 | 82/52.5 | 10/17 | Y/N | DSM-5 | Plasma | NA | 19/18 | Hospital |
| Musthafa et al. 2011 | leptin | Sultan Qaboos， Oman | 19/19 | 78.9/52.6 | NA | Y/N | DSM-IV-TR | Plasma | NA | NA | Family |
| Quan et.al 2021 | adiponectin | Nanchang, China | 88/88 | 77.3/77.3 | 4.3/4.3 | Y/Y | DSM-5 | Serum | ELISA | 20.5/20.7 | Kindergarten |
| Özgeriş et.al 2022 | progranulin | Ataturk, Turkey | 20/35 | 20/75 | 3.51/3.88 | Y/N | DMS-5 | Serum | ELISA | NA | NA |
| Raghavan et al. 2018 | leptin | Boston, USA | 36/ 616 | 73.58/ 42.52 | 30.35/28.21 | Y/N | ICD-9 | Plasma | Sandwich immunoassay | NA | NA |
| Rodrigues et al. 2014 | adiponectin; leptin; resistin | Belo Horizonte, Brazil | 30/19 | NA | NA | N/N | DSM-IV-TR | Plasma | ELISA | NA | Hospital |
| Sadıç et al. 2021 | ghrelin; leptin | Konya, Turkey | 44/44 | 86.36/79.55 | 2.86/3.04 | Y/Y | DSM-V | Plasma | ELISA | 44.6/31.3 | Hospital |

**Abbreviations**: ASD, Autistic Spectrum Disorder; HC, Healthy Control; Y/N, yes/no; BMI, Body Mass Index.

**eTable 2.** Newcastle-Ottawa quality assessment scale for included studies.

| **Between-Group Studies** | **Selection** | **Comparability** | **Exposure** | **Total** |
| --- | --- | --- | --- | --- |
| AL-ayadhi et.al 2011 | *** | * | ** | 6 |
| Al-Zaid et al. 2014 | *** | ** | ** | 7 |
| Ashwood et al. 2008 | **** | * | ** | 7 |
| Blardi et al. 2009 | *** | * | * | 5 |
| Blardi et al. 2010 | ** | ** | ** | 6 |
| Chen et al. 2023 | *** | ** | ** | 8 |
| Frenssen et.al 2015 | *** | ** | ** | 7 |
| Fujita-Shimizu et.al 2010 | ** | ** | * | 5 |
| Ghaffari et.al 2016 | **** | * | ** | 7 |
| Jarmołowska et.al 2019 | *** | * | * | 5 |
| Lisik et al. 2016 | **** | * | ** | 7 |
| Maekawa et al. 2020 | *** | ** | * | 6 |
| Manco et.al 2021 | * | ** | ** | 5 |
| Musthafa et al. 2011 | ** | ** | * | 5 |
| Quan et.al 2021 | **** | ** | ** | 8 |
| Özgeriş et.al 2022 | *** | ** | ** | 7 |
| Raghavan et al. 2018 | *** | ** | ** | 7 |
| Rodrigues et al. 2014 | *** | ** | ** | 7 |
| Sadıç et al. 2021 | ** | * | * | 4 |

**eFigure: Funnel plots for leptin.**

Funnel plots for studies analyzing serum leptin levels

**eReference:**

1. Al-ayadhi LY, Mostafa GA. Increased serum osteopontin levels in autistic children: relation to the disease severity. Brain Behav Immun. 2011 Oct;25(7):1393-8. doi: 10.1016/j.bbi.2011.04.006.
2. Al-Zaid FS, Alhader AA, Al-Ayadhi LY. Altered ghrelin levels in boys with autism: a novel finding associated with hormonal dysregulation. Sci Rep. 2014 Sep 26;4:6478. doi: 10.1038/srep06478.
3. Ashwood P, Kwong C, Hansen R, Hertz-Picciotto I, Croen L, Krakowiak P, Walker W, Pessah IN, Van de Water J. Brief report: plasma leptin levels are elevated in autism: association with early onset phenotype? J Autism Dev Disord. 2008 Jan;38(1):169-75. doi: 10.1007/s10803-006-0353-1.
4. Blardi P, de Lalla A, D'Ambrogio T, Vonella G, Ceccatelli L, Auteri A, Hayek J. Long-term plasma levels of leptin and adiponectin in Rett syndrome. Clin Endocrinol (Oxf). 2009 May;70(5):706-9. doi: 10.1111/j.1365-2265.2008.03386.x.
5. Blardi P, de Lalla A, Ceccatelli L, Vanessa G, Auteri A, Hayek J. Variations of plasma leptin and adiponectin levels in autistic patients. Neurosci Lett. 2010 Jul 19;479(1):54-7. doi: 10.1016/j.neulet.2010.05.027.
6. Frenssen F, Croonenberghs J, Van den Steene H, Maes M. Prolyl endopeptidase and dipeptidyl peptidase IV are associated with externalizing and aggressive behaviors in normal and autistic adolescents. Life Sci. 2015 Sep 1;136:157-62. doi: 10.1016/j.lfs.2015.07.003.
7. Fujita-Shimizu A, Suzuki K, Nakamura K, Miyachi T, Matsuzaki H, Kajizuka M, Shinmura C, Iwata Y, Suda S, Tsuchiya KJ, Matsumoto K, Sugihara G, Iwata K, Yamamoto S, Tsujii M, Sugiyama T, Takei N, Mori N. Decreased serum levels of adiponectin in subjects with autism. Prog Neuropsychopharmacol Biol Psychiatry. 2010 Apr 16;34(3):455-8. doi: 10.1016/j.pnpbp.2009.12.020.
8. Ghaffari MA, Mousavinejad E, Riahi F, Mousavinejad M, Afsharmanesh MR. Increased Serum Levels of Tumor Necrosis Factor-Alpha, Resistin, and Visfatin in the Children with Autism Spectrum Disorders: A Case-Control Study. Neurol Res Int. 2016;2016:9060751. doi: 10.1155/2016/9060751.
9. Jarmołowska B, Bukało M, Fiedorowicz E, Cieślińska A, Kordulewska NK, Moszyńska M, Świątecki A, Kostyra E. Role of Milk-Derived Opioid Peptides and Proline Dipeptidyl Peptidase-4 in Autism Spectrum Disorders. Nutrients. 2019 Jan 4;11(1):87. doi: 10.3390/nu11010087.
10. Lisik MZ, Gutmajster E, Sieroń AL. Plasma Levels of Leptin and Adiponectin in Fragile X Syndrome. Neuroimmunomodulation. 2016;23(4):239-243. doi: 10.1159/000452336.
11. Maekawa M, Ohnishi T, Toyoshima M, Shimamoto-Mitsuyama C, Hamazaki K, Balan S, Wada Y, Esaki K, Takagai S, Tsuchiya KJ, Nakamura K, Iwata Y, Nara T, Iwayama Y, Toyota T, Nozaki Y, Ohba H, Watanabe A, Hisano Y, Matsuoka S, Tsujii M, Mori N, Matsuzaki H, Yoshikawa T. A potential role of fatty acid binding protein 4 in the pathophysiology of autism spectrum disorder. Brain Commun. 2020 Sep 10;2(2):fcaa145. doi: 10.1093/braincomms/fcaa145.
12. Manco M, Guerrera S, Ravà L, Ciofi Degli Atti M, Di Vara S, Valeri G, Vicari S. Cross-sectional investigation of insulin resistance in youths with autism spectrum disorder. Any role for reduced brain glucose metabolism? Transl Psychiatry. 2021 Apr 20;11(1):229. doi: 10.1038/s41398-021-01345-3.
13. Musthafa ME, et al. Elevated Plasma Leptin Levels in Autistic Children of Sultanate of Oman. International Journal of Biological & Medical Research. 2011;2:803-805.
14. Quan L, Zhao Y, Yi J, Shi XD, Zhong Y, Liu L. Serum adiponectin levels are reduced in autism spectrum disorder and association with severity of symptoms. Metab Brain Dis. 2021 Mar;36(3):491-498. doi: 10.1007/s11011-020-00668-2.
15. Özgeriş FB, Kurt N, Ibili Ucuz I, Koçak Yilmaz K, Keleş MS, Çayir A, Dursun OB. Is Serum Progranulin Level a Biomarker in Autism and Cognitive Development Disorders? Eurasian J Med. 2022 Feb;54(1):50-53. doi: 10.5152/eurasianjmed.2022.21292.
16. Raghavan R, Zuckerman B, Hong X, Wang G, Ji Y, Paige D, DiBari J, Zhang C, Fallin MD, Wang X. Fetal and Infancy Growth Pattern, Cord and Early Childhood Plasma Leptin, and Development of Autism Spectrum Disorder in the Boston Birth Cohort. Autism Res. 2018 Oct;11(10):1416-1431. doi: 10.1002/aur.2011.
17. Rodrigues DH, Rocha NP, Sousa LF, Barbosa IG, Kummer A, Teixeira AL. Changes in adipokine levels in autism spectrum disorders. Neuropsychobiology. 2014;69(1):6-10. doi: 10.1159/000356234.
18. Çelikkol Sadıç Ç, Bilgiç A, Kılınç İ, Oflaz MB, Baysal T. Evaluation of Appetite-Regulating Hormones ın Young Children with Autism Spectrum Disorder. J Autism Dev Disord. 2021 Feb;51(2):632-643. doi: 10.1007/s10803-020-04579-0.
